# Generalized Morphea after Breast Cancer Radiation Therapy

**DOI:** 10.1155/2011/951948

**Published:** 2011-11-03

**Authors:** Jonathan Kushi, M. E. Csuka

**Affiliations:** Department of Rheumatology, Medical College of Wisconsin, FEC 4764, 9200 W. Wisconsin Avenue, Milwaukee, WI 53226, USA

## Abstract

We present a case of a 69-year-old woman who received external beam radiation for the treatment of breast cancer. Seven months later, she developed generalized morphea involving the area of irradiated skin of the breast as well as distant sites of the groin and distal lower extremity. Postirradiation morphea is an uncommon yet well-documented phenomenon, usually confined to the radiated site and the immediate surrounding tissue. To our knowledge, this is only the fourth reported case of morphea occurring distant from the radiation field. While most cases of postirradiation morphea have been shown to either resolve spontaneously or respond to topical corticosteroids, our patient required systemic therapy with methotrexate, which resulted in clinical improvement. With this paper, we hope to bring further awareness to this phenomenon and demonstrate a successful treatment response with the use of methotrexate in postirradiation generalized morphea.

## 1. Introduction

Morphea is a rare, idiopathic, chronic inflammatory disease of the skin and underlying tissues causing fibrosis of the skin, subcutaneous tissue, and in some cases the underlying fascia, muscle, or bone. It typically begins as an erythematous or violaceous plaque or patch that becomes indurated as sclerosis develops over time. Morphea is also known as localized scleroderma. It is differentiated from systemic sclerosis by the absence of internal organ involvement, sclerodactyly, nail-fold capillary changes, and Raynaud's phenomenon. There is no clear consensus regarding the classification of morphea. One of the more recognized classification systems, proposed by Laxer and Zulian, is based on clinical presentation and describes five subtypes: circumscribed (superficial and deep), linear (trunk/limb, en coup de sabre, Parry-Romberg), generalized, pansclerotic and mixed variants [1, 2].

Although the specific etiology of morphea is unknown, several factors are recognized as triggers, including traumatic injury, infection, chemical, and radiation exposure. Morphea following radiation exposure is well documented [3–5]. In contrast to idiopathic morphea with a reported incidence of 2.7 per 100,000 in the United States [6, 7], the incidence of postirradiation morphea has been estimated to be as high as 1 in every 500 patients [8, 9]. It is most frequently described in patients who received focal radiotherapy for breast cancer [3–5, 8–10] but is reported as a complication of radiation therapy for lymphoma [11], head and neck cancer [12, 13], and gastric [14], endocervical [8, 12], and endometrial [15, 16] cancers. For the majority of cases, the area of involved skin is confined to the field of radiation exposure or nearby surrounding tissue. Onset is usually within the first year, but development of postirradiation morphea has been described as late as 37 years [3]. The early morphea lesion may have a similar appearance and must be distinguished from sclerosing carcinoma, cellulitis, or radiation dermatitis. Histologically, morphea is defined initially by early lymphocytic perivascular inflammation that in later stages shows progression to collagen-bundle hypertrophy.

We report a case of generalized morphea following local irradiation for treatment of breast cancer. We will review the existing literature and discuss some of the various approaches to treatment. 

## 2. Case Report

A 69-year-old woman presented to the Rheumatology Clinic in January 2010 for evaluation of generalized morphea/localized scleroderma diagnosed 7 months after receiving local radiation to right breast for treatment of breast cancer. In September 2008, breast cancer was diagnosed by needle biopsy when an abnormal cluster of calcifications was identified by a screening mammography in September 2008. After consultation with her surgeon, oncologist, and radiation therapist, she underwent lumpectomy and sentinel lymph node biopsy. The cancer was estrogen receptor negative, and the sampled lymph nodes were negative. The tumor was estrogen and progesterone receptor positive. She opted not to take tamoxifen or other antiestrogen therapy. To prevent cancer recurrence, external beam radiation to right breast was completed (October 2008). She experienced local radiation side effects of erythema and superficial blister formation that resolved with local wound care.

Approximately 7 months after completing radiation therapy, she developed two painful blisters in the inframammary fold of the right breast. On examination she was found to have generalized induration of the right breast and right axilla attributed to postradiation fibrosis. The breast and axilla were warm to touch and painful to palpation. That was with sparing of the areola. Additional lesions distant from the radiation field were also noted. These included hyperpigmentation of the skin at the waistline, induration of the skin in the left upper inner thigh and left groin, and a shiny patch of thickened skin on the anterior left shin. The lesions not involving the breast and axilla were not painful.

In May 2009, a punch biopsy of the left groin lesion diagnosed localized scleroderma (morphea). On microscopy, the skin surface was thin with a slightly keratotic epidermis. The papillary dermis was edematous and homogenous. The reticular dermis had dense collagen in bundles with intervening mild lymphoid and plasma cellular inflammation present around the adnexal structures. Mild chronic inflammation in the subcutaneous fat was noted at the base of the biopsy. For treatment of the right breast and axillary lesions, topical calcipotriol and topical betamethasone dipropionate were prescribed without benefit. Painful ulcers developed in the area of application in the right axilla which healed slowly when the topical medications were discontinued. The area under the right breast remained painful with chronic superficial ulceration. Application of topical silver impregnated dressings was of no benefit.

In addition to the skin lesions, she described fatigue and generalized arthralgias. She denied more specific symptoms of systemic connective tissue disease, that is, no history of Raynaud's, esophageal reflux, shortness of breath, cough, joint swelling, morning stiffness, sicca symptoms, pleuritis, or serositis. Prior to diagnosis of breast cancer, she had no significant past medical or psychiatric history and was on no prescribed medications. Family history was negative for connective tissue disease. Physical examination confirmed circumferential induration of the right breast with sparing of the areola. Induration extended into the right axilla. Superficial erosion of the epidermis was noted in the inframammary fold of the right breast. A band of hyperpigmented, thickened skin was noted along the anterior waistline. A hypopigmented indurated patch was noted in the left groin with a healed punch biopsy site. Thinning of the skin with hypopigmentation and superficial telangiectasias was noted in the upper left medial thigh. A 3 cm patch with central hypopigmentation was present on the left anterior mid tibia. She had no sclerodactyly. Nail fold examination for capillary changes was normal. Examination of the heart, lungs, and abdomen was normal. Laboratory testing was normal, including complete blood count, chemistry panel, thyroid function tests, and urine analysis. Sedimentation rate and C-reactive protein were normal. Lyme titer was negative. Thyroid autoantibody testing was negative. ANA was interpreted as borderline positive, with titer of 1 : 160 in a speckled pattern. Further testing for ANA subtypes was negative for anti-topoisomerase, anti-RNP, anti-Smith, anti-ds DNA, anti-SSA, and anti-SSB.

She was prescribed minocycline 100 mg twice a day, and, after three days of therapy, she reported dramatic improvement in generalized pain and fatigue. The superficial erosions in the inframammary fold healed. However, despite the initial perceived benefit, the right breast remained warm and tender to palpation. New lesions occurred on the right lower extremity. In August 2010, methotrexate 7.5 mg weekly and titrated to 12.5 mg was prescribed. Folic acid 1 mg per day was prescribed to prevent methotrexate side effects. After the addition of methotrexate, no new lesions occurred. Signs of inflammation in the irradiated right breast resolved though skin thickening remained unchanged. The lesions on bilateral lower extremities softened. Minocycline was discontinued in March 2011 when abnormal pigmentation developed as a side effect. She remained without signs or symptoms of a systemic connective tissue disease.

## 3. Discussion

Radiation-induced dermatologic manifestations are common with as many as 90% of patients reporting some type of local skin reaction [17]. The changes that occur early, within the first two months of exposure, are consistent with local irritation and drying of the skin secondary to inflammatory cytokine production [18]. This can cause hyperpigmentation, hair loss, or desquamation of the surrounding skin. Late effects, defined as 2 months up to decades after last radiation exposure, include fibrosis, telangiectasias and skin necrosis. The incidence of late-stage reactions has been reported in up to 30% of cases [19]. 

Postirradiation morphea was described as early as 1905 following exposure to radiographs [20]. However, it was not until 1989 that it was recognized as a complication of radiotherapy for cancer when Colver et al. [10] reported their series of 9 cases of which 7 were patients treated for breast cancer. A recent review of the published literature cited 42 cases of postirradiation morphea documented since 1989 [21]. All but six of these cases occurred in the setting of breast cancer. The increased occurrence of postirradiation morphea in breast cancer may relate to the deliberate inclusion of the cutis and subcutaneous tissue in the area to be treated. 

An increase risk of adverse effects after radiotherapy in patients with known collagen vascular disease has been a concern since the 1970s. Patients with collagen vascular disease diagnosed with early-stage breast cancer may not be offered breast conservation therapy which includes radiotherapy even when signs and symptoms of the collagen vascular disorder are quiescent [22]. In the published cases of postirradiation morphea, no predictable risk factors have been identified. There is no apparent relation to radiation dose [10]. There is speculation that patients with generalized morphea are more likely to have positive serology for autoantibodies, especially ANA [23]. Our patient did have a positive ANA, at borderline titer of 1 : 160, speckled pattern. However, she had no other clinical findings of systemic sclerosis, that is, no evidence of internal organ involvement, sclerodactyly, nail fold capillary changes or Raynaud's. 

Patients with systemic sclerosis have been reported to have an exaggerated radiation-induced fibrotic response [24]. In postirradiation morphea, it has been proposed that radiation exposure may activate clonal fibroblasts resulting in increased cytokine production, such as transforming growth factor-*β* (TGF-*β*) [8]. This cytokine has been implicated in the pathophysiology of both localized and diffuse scleroderma [25, 26]. Analysis of skin biopsy sections have shown increased staining for TGF-*β* in limited scleroderma, morphea and diffuse cutaneous scleroderma. The increased cytokine response is believed to result in increased glycosaminoglycan production, collagen synthesis and extracellular matrix protein secretion [8, 27].

The majority of reported postirradiation morphea cases would be classified as circumscribed morphea with lesions confined to the radiated field and surrounding tissue [5, 21]. Our case is unusual as it involved both the irradiated breast as well as skin not exposed to radiation, that is, the groin and distal lower extremities. Her condition is classified as generalized morphea defined as four or more indurated plaques on more than two of seven anatomic sites [2]. To date, only three previous case reports of postirradiation morphea with involvement of skin distant from the areas of focal radiation are reported [15, 16, 28]. Interestingly, all of these cases involved lesions of the lower extremities. Our patient had a fairly synchronous onset of her skin findings, which is similar to the case described by Ardern-Jones and Black [28]. In the case reported by Ullén and Björkholm, the patient had received radiotherapy for two unrelated cancers. She was first treated for endometrial cancer with intravaginal irradiator followed by external beam pelvic radiation after total hysterectomy and bilateral salpingo-oophorectomy. Three years later she had partial mastectomy and post surgical radiation for treatment of stage I breast cancer. Morphea of the irradiated breast occurred 2 months after completing radiotherapy. A year later an abdominal wall lesion was noted in the area irradiated for treatment of endometrial cancer. Then 5 and 8 years after prior radiotherapies, she developed morphea in the lower leg, [15]. The third case described linear scleroderma beginning in the irradiated pelvic region and extending to the lower extremity [16]. 

Given the limited number of cases reported, response to therapy is not well defined. The natural history of these cases is highly variable with many resolving spontaneously without intervention or with topical glucocorticoids [3, 21]. Other approaches to treatment include methotrexate with and without systemic glucocorticoids, psoralen with ultraviolet radiation and surgical excision [29]. 

Our patient had a poor response to combination topical glucocorticoid and calcipotriol. She was opposed to systemic immunosuppressive therapy. Based on anecdotal reports, she agreed to a trial of minocycline with initial encouraging response describing immediate improvement in generalized fatigue and healing of superficial erosions under the breast. However, she developed new lesions on the right lower extremity. After taking methotrexate, no new lesions developed, and established lesions softened and regressed. The methotrexate dose of 12.5 mg per week was considerably higher than 2.5 mg per week used in the case reported by Ardern-Jones and Black [28]. 

Given the unpredictable nature of this disease and the rare incidence, it is difficult to establish standardized approaches to treatment. Further research into the pathogenesis of postirradiation morphea may provide additional insight into the risk factors and prognostic indicators of this disease. With increased awareness of this syndrome, we may also have a better understanding of the patterns of treatment response. This may help to establish future management strategies in choosing between local or systemic therapy depending on the extent of skin involvement.
